# Maxillary Swelling as the First Evidence of Multiple Myeloma

**DOI:** 10.1155/2015/439536

**Published:** 2015-11-12

**Authors:** Atsushi Kasamatsu, Yasushi Kimura, Hideki Tsujimura, Harusachi Kanazawa, Nao Koide, Isao Miyamoto, Yosuke Endo-Sakamoto, Masashi Shiiba, Hideki Tanzawa, Katsuhiro Uzawa

**Affiliations:** ^1^Department of Dentistry and Oral-Maxillofacial Surgery, Chiba University Hospital, 1-8-1 Inohana, Chuo-ku, Chiba 260-8670, Japan; ^2^Department of Oral Science, Graduate School of Medicine, Chiba University, 1-8-1 Inohana, Chuo-ku, Chiba 260-8670, Japan; ^3^Division of Clinical Oncology, Chiba Cancer Center, 666-2 Nitona, Chuo-ku, Chiba 260-8717, Japan; ^4^Division of Dentistry and Oral and Maxillofacial Surgery, Sammu Medical Center, 167 Naruto, Sammu, Chiba 289-1326, Japan; ^5^Department of Clinical Oncology, Graduate School of Medicine, Chiba University, 1-8-1 Inohana, Chuo-ku, Chiba 260-8670, Japan

## Abstract

Multiple myeloma is a malignant neoplasm of plasma cells characterized by proliferation of a single clone of abnormal immunoglobulin-secreting plasma cells. Since the amount of hemopoietic bone marrow is decreased in the maxilla, oral manifestations of multiple myeloma are less common in the maxilla than in the mandible. We report the case of 33-year-old Japanese man who presented with a mass in the right maxillary alveolar region. Computed tomography and magnetic resonance images showed a soft tissue mass in the right maxilla eroding the anterior and lateral walls of the maxillary sinus and extending into the buccal space. The biopsy results, imaging, and laboratory investigations led to the diagnosis of multiple myeloma. This case report suggests that oral surgeons and dentists should properly address oral manifestations as first indications of multiple myeloma.

## 1. Introduction

Multiple myeloma, a malignant neoplasm of plasma cells, is the most common primary bone malignancy characterized by monoclonal proliferation of plasma cells [1, 2]. The diagnosis of multiple myeloma usually is confirmed by abnormal immunoglobulin (M-protein) in the serum or urine and/or multiple punched-out radiolucent lesions together with histologic confirmation of malignant growth of plasma cells [3]. The clinical manifestations of the disease occur due to an expanding mass of plasma cells in the bone marrow [4]. The most common clinical signs and symptoms of multiple myeloma include renal failure, bone pain, fatigue, anemia, hypercalcemia, and infectious diseases [4, 5].

Oral manifestations of multiple myeloma very rarely present as the initial signs [6]. Few case reports of multiple myeloma with maxillary involvement have been published. We describe a case of multiple myeloma involving the maxilla in a 33-year-old man who experienced swelling with mobility of the teeth in the right maxillary alveolar region.

## 2. Case Report

**Figure 1:**
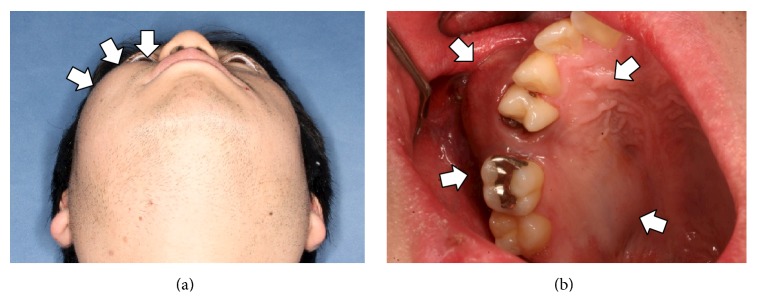
Extraoral (a) and intraoral (b) photographs show diffuse swelling in the right cheek and maxillary alveolar region (arrows).

**Figure 2:**
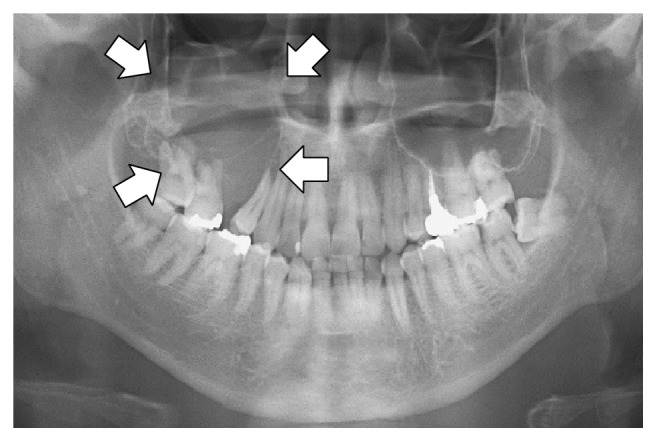
Panoramic radiography shows an osteolytic lesion in the right posterior maxilla with resorption of the floor of the maxillary sinus (arrows).

**Figure 3:**
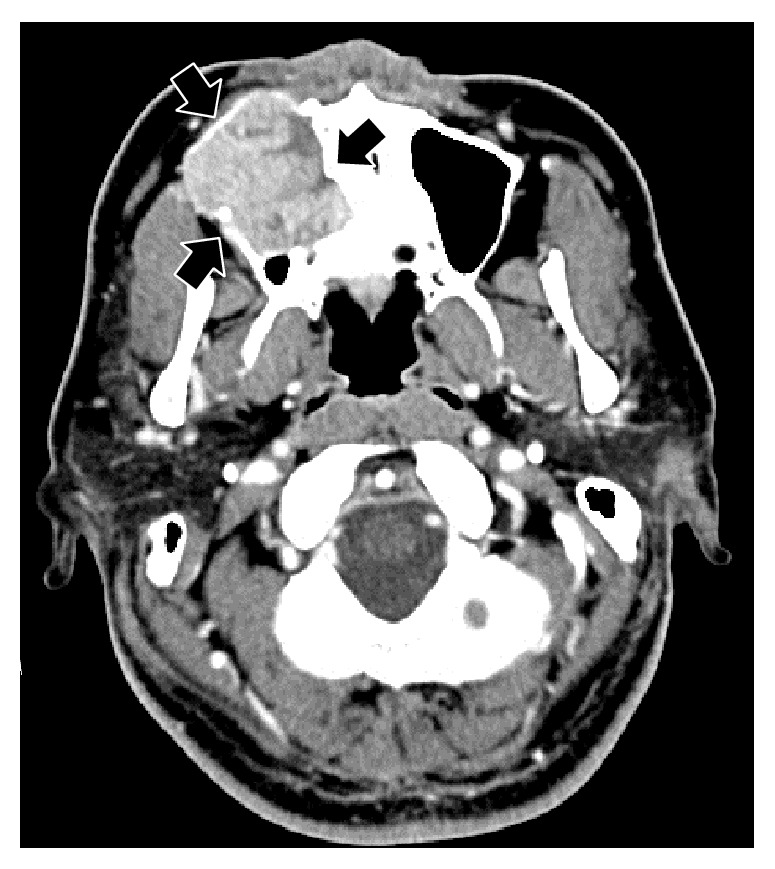
An axial CT image shows a soft tissue density mass eroding the anterior and lateral walls of right maxillary sinus (arrows).

**Figure 4:**
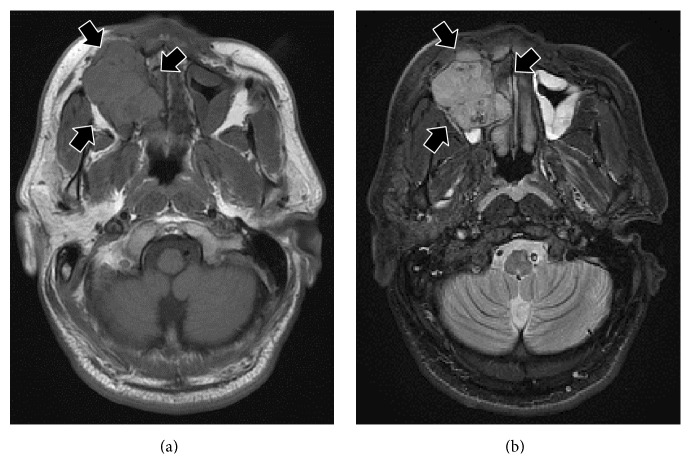
An MRI scan shows a mass with low T1- (a) and moderate T2-weighted (b) signal intensity.

**Figure 5:**
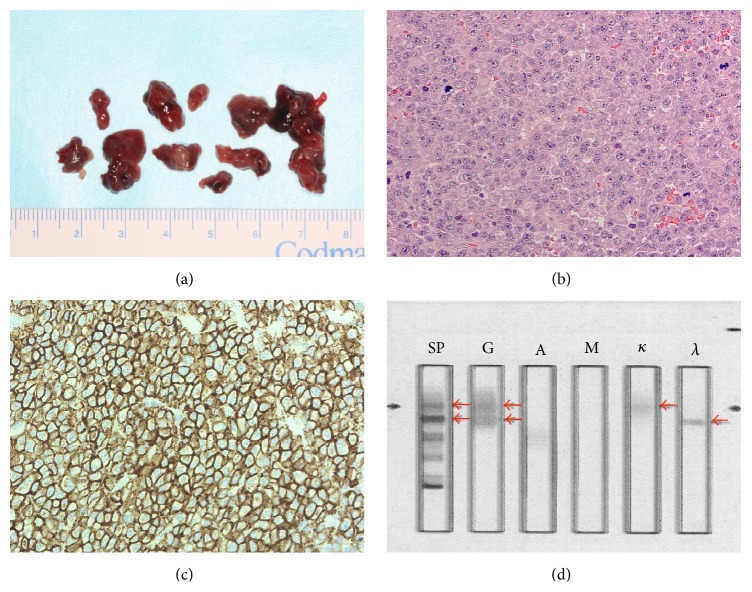
An incisional biopsy specimen from the maxillary lesion (a). Hematoxylin and eosin staining of the specimen shows solid proliferation of plasmacytoid cells with eccentric nuclei and basophilic cytoplasm and partially shows atypia ((b), original magnification ×40). The immunohistochemical results are positive for CD138 ((c), original magnification ×40). Serum immunofixation electrophoresis shows M-protein (IgG *κ* and *λ* types, (d)).

**Figure 6:**
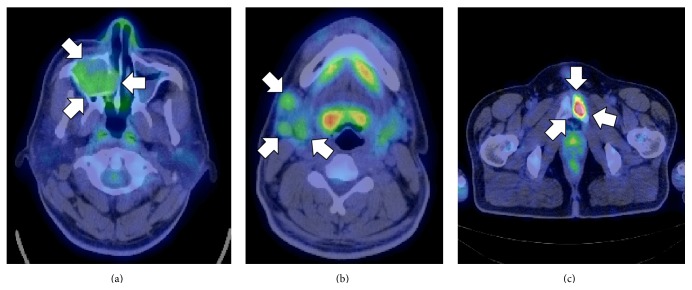
Strong SUVs are found in the right maxillary sinus ((a), arrows), right submandibular region ((b), arrows), and left pubic bone ((c), arrows) on an ^18^F-FDG PET/CT scan.

A 33-year-old Japanese man presented with diffuse swelling in the right cheek and maxillary alveolar region with mobility of the adjacent teeth, the first premolar and first and second molars (Figure 1). The upper left second premolar had been extracted 10 years previously due to dental root fracture. A soft, nonpulsatile, nontender, and nonhemorrhagic intraoral mass extended from the right first premolar to the tuberosity region. A panoramic radiograph showed an ill-defined osteolytic lesion in the right posterior maxilla with resorption of the floor of the maxillary sinus (Figure 2). An axial section computed tomography (CT) image showed a soft tissue density mass measuring 45 × 35 mm in the right maxilla eroding the anterior and lateral walls of the maxillary sinus and extending into the buccal space (Figure 3). Magnetic resonance imaging (MRI) also showed a mass with low T1- and moderate T2-weighted (Figure 4) signal intensity in the right maxilla. After the radiographic evaluations, an incisional biopsy of the maxillary mass was performed; histopathologic examination of the specimen showed atypical plasma cells with large hyperchromatic nuclei, binucleation, and large cytoplasms (Figures 5(a) and 5(b)). Immunohistochemically, the cells were positive for CD138 (Figure 5(c)). Laboratory analysis showed increased immunoglobulin (IgG, 2.33 g/dL) levels. No Bence-Jones protein was present in the urine. Serum immunofixation electrophoresis showed M-protein (IgG *κ* and *λ* types, Figure 5(d)). However, no signs of renal failure or hypercalcemia and abnormal signs of fluorescence in situ hybridization were found. An ^18^F-fluorodeoxyglucose positron emission CT (^18^F-FDG PET/CT) image showed a strong standardized uptake value (SUV) in the right maxillary sinus, right submandibular region, and left pubic bone (Figure 6). Based on the clinical data, a final diagnosis of multiple myeloma was made. Consequently, the patient was referred for treatment to the Division of Clinical Oncology, Chiba Cancer Center. The patient underwent bortezomib plus dexamethasone chemotherapy in the hospital and has tolerated the chemotherapy well until now.

## 3. Discussion

Multiple myeloma accounts for 1% of all malignancies and slightly more than 10% of hematologic malignancies [5]. The diagnosis of multiple myeloma depends on the identification of abnormal monoclonal plasma cells and the results of a full blood count, bone marrow biopsy, M-protein levels in the serum or urine, and clinical images consistent with multiple myeloma [7]. Bone marrow examination showed a large amount of these abnormal plasma cells, which produce M-protein light chain proteins (*κ* and *λ* types), and cytokines. In the current case, serum protein electrophoresis showed an IgG monoclonal spike of 2.33 g/dL with the *κ* and *λ* light chains. Excessive production of M-protein causes serum hyperviscosity, which in turn leads to renal dysfunction [8, 9]. Although urine electrophoresis may identify M-protein in 60% of patients [10, 11], we did not detect M-protein in the urine. Myeloma cells are typically positive for CD138 [2], which is a useful molecule for determining the extent of plasma cellular infiltration that expresses immunoglobulin in the cytoplasm and occasionally on the cellular surface. Therefore, immunohistochemistry is critical to confirm multiple myeloma [12].

Multiple, well-defined, punched-out radiolucencies without a definitive cortical margin that often contain abnormal plasma cellular proliferations are radiographic features of multiple myeloma [13]. ^18^F-FDG PET/CT scans can visualize focal alterations of the bone marrow before punched-out radiolucencies are detected by conventional radiographs [14]. Whereas the current case did not have punched-out radiolucent lesions, ^18^F-FDG PET/CT showed multiple strong SUVs in the right maxillary sinus, right submandibular region, and left pubic bone, indicating that ^18^F-FDG PET/CT is a helpful tool for diagnosing multiple myeloma.

Treatment of multiple myeloma involves mainly irradiation, chemotherapy, and autologous stem cell transplantation. Prognosis is determined via risk classification by the International Staging System (ISS), which defines three risk categories using serum concentrations of *β*2 microglobulin and albumin [2, 15]. In the current case, because both concentrations were low, the current case was classified as ISS stage I with a median survival period of 62 months. Our patient underwent bortezomib plus dexamethasone chemotherapy and has tolerated the chemotherapy well until now.

Oral manifestations of multiple myeloma rarely present as the first signs [13, 14, 16–18]; the clinical features are swelling, orofacial pain, mobility of teeth, paresthesia, hemorrhage, fracture, and root resorption [13, 19]. The maxilla and mandible are the more common regions at which multiple myeloma occurs, with incidence rates ranging from 8% to 15% among the regions of oral manifestations [20]. Since maxillary swelling and mobility of the maxillary molars were the first signs in the current case, carcinoma of the maxillary sinus and metastatic carcinoma from other organs should be considered in the differential diagnosis.

In summary, we reported a rare case of multiple myeloma presenting with swelling in the maxilla as a first sign and suggest that oral surgeons and dentists should properly address oral manifestations as first indications of multiple myeloma.
